# Pediatric hip disorders are not associated with an increased 10-year revision risk after total hip arthroplasty under the age of 55: results from the Dutch Arthroplasty Register

**DOI:** 10.2340/17453674.2024.41342

**Published:** 2024-08-28

**Authors:** Michaël P A BUS, Maaike G J GADEMAN, Marta FIOCCO, Rob G H H NELISSEN, Pieter Bas DE WITTE

**Affiliations:** 1 Department of Orthopedic Surgery, Leiden University Medical Center, Leiden; 2 Department of Clinical Epidemiology, Leiden University Medical Center, Leiden; 3 Department of Biomedical Data Science, section Medical Statistics, Mathematical Institute Leiden University, Leiden, The Netherlands

## Abstract

**Background and purpose:**

Developmental dysplasia (DDH) and Legg–Calvé–Perthes disease (LCPD) are common indications for total hip arthroplasty (THA) at a young age, and may be associated with increased revision risk. We aimed to investigate the 10-year cumulative aseptic cup revision and overall revision risk of THA, and investigated whether these are increased compared with THA for primary osteoarthritis (OA) in patients below 55 years.

**Methods:**

All THAs (2007–2019) in patients under the age of 55 for the indications OA, DDH, and LCPD were extracted from the Dutch Arthroplasty register. The 10-year cumulative incidences of aseptic cup failure and overall revision were assessed for the 3 groups, with death as a competing risk. Cox regression analysis was used.

**Results:**

24,263 THAs were identified: 20,645 (85%) for OA, 3,032 (13%) for DDH, and 586 (2%) for LCPD. The 10-year cumulative revision risk for aseptic cup failure was 3.4% (95% confidence interval [CI] 3.0–3.8) for OA, 3.4% (CI 2.4–3.4) for DDH, and 1.7% (CI 0.2–3.1) for LCPD. The 10-year cumulative overall revision risk was 6.0% (CI 5.6–6.5) for OA, 6.0% (CI 4.9–7.2) for DDH, and 5.1% (2.7–7.5) for LCPD. The multivariable Cox regression analysis for aseptic cup failure yielded hazard ratios of 0.7 (0.5–1.2) for DDH, and 0.8 (0.3–2.1) for LCPD compared with OA. No statistically significant differences for overall revision were found.

**Conclusion:**

THA performed for DDH or LCDP in patients under the age of 55 was not associated with a statistically significant increased risk of aseptic cup revision or overall revision, compared with THA performed for primary OA in the same age group.

The cumulative 10-year revision risk after total hip arthroplasty (THA) is around 1.7% to 5.3% and is increased in younger patients [1]. The number of hip arthroplasties in younger patients is increasing, and it is plausible that revision risk is higher in younger patients with THA for secondary osteoarthritis due to pediatric hip disorders, resulting from, for example, pathomorphological changes or limited bone stock [2].

Most studies on THA in younger patients have not stratified per underlying diagnosis or focused on the outcomes of THA for 1 specific indication [3-6]. However, the indications for THA in younger patients are skewed towards pediatric hip disorders, including developmental dysplasia of the hip (DDH) or Legg–Calvé–Perthes disease (LCPD), which are important causes of secondary osteoarthritis. As these disorders often result in limited bone stock as well as multiplanar pathomorphological changes, THA in these patients is often technically challenging [7,8]. In dysplastic hips, acetabular reconstruction is usually the most complex, as the cup should be placed at the anatomic hip center while bone stock is commonly deficient, with a small-diameter native acetabulum, increased femoral anteversion, and coxa valga [7]. On the other hand, LCPD often leads to deformities on both the femoral and acetabular side. These include enlargement and flattening of the femoral head (making it difficult to obtain adequate exposure), increased anteversion of the femoral neck, and a shallow, lateralized, and retroverted acetabulum [9]. In addition, patients with DDH and LCPD are more likely to have undergone previous surgical procedures before THA and that in itself may lead to surgical challenges as a result of osseous deformities and soft tissue scarring. Although a higher complication risk may be expected, conflicting results have been reported [10,11].

The aim of our study was to assess whether THAs performed below the age of 55 for secondary osteoarthritis (OA) due to pediatric hip disorders (DDH or LCPD) have an increased the 2-, 5-, and 10-year risk of aseptic cup failure or overall revision, compared with THA for primary OA.

## Methods

### Study design and data source

We obtained data from the LROI on all THAs from 2007 to 2019 in patients under the age of 55, for the registered indications primary OA, or secondary OA due to a history of DDH or LCPD. The reported completeness for primary THA was over 95% up to 2013 [12], and further increased to 99% in 2020 (www.lroi-report.nl). For hip revision arthroplasties, the completeness was 88% in 2013, and this increased to 97–98% between 2015 and 2022 (www.lroi-report.nl). Diagnoses were not validated.

The study is reported according to STROBE guidelines.

### Data selection

Hemiarthroplasties, metal-on-metal THAs and THA for other indications, including rheumatoid arthritis, a (pathological) fracture, osteonecrosis, primary or metastatic tumor, late post-traumatic changes, inflammatory arthritis, or other reasons, were excluded. Minimum follow-up was 2 years.

### Variables

The following data were extracted from the register: age at surgery (years), patient sex (male/female), joint side (left/right), American Society of Anesthesiologists (ASA) classification, previous surgery on index joint (yes/no), body mass index (BMI), smoking status at time of surgery (yes/no), indication for surgery (OA, DDH or LCPD), implant fixation type (uncemented, cemented, hybrid [uncemented cup and cemented stem], or reverse hybrid [cemented cup and uncemented stem]), surgical approach (direct anterior, anterolateral, straight lateral, posterolateral and other), bearing type (zirconium on polyethylene [ZoP], metal on polyethylene [MoP], ceramic-on-polyethylene [CoP] and ceramic-on-ceramic [CoC]), femoral head and cup size used, and time between the index procedure and revision, end of follow-up, or death.

### Outcomes

In the LROI, only revision procedures are registered in which 1 or more prosthetic components are exchanged. Aseptic cup survival was defined as the time from the index procedure to revision of the cup in the absence of periprosthetic joint infection. Overall survival was defined as the time from THA to any revision procedure in which the stem or cup was removed or replaced, i.e., revision for any reason.

### Statistics

Baseline characteristics were summarized by using means and standard deviations (SD) or medians and ranges for continuous variables; frequencies and percentages were applied for categorical variables. Where applicable, 95% confidence intervals (CI) are provided. Competing risks models were employed to estimate the cumulative incidence of cup failure for aseptic reasons and of overall revision for the 3 different diagnoses, with death as a competing event [13,14]. Cox regression models (adjusted for patient sex, previous surgery, age, BMI, and acetabular cup size—the latter 3 as continuous variables) were used to estimate the cause-specific hazard ratios of the underlying diagnosis on revision risk within the first 10 years, with endpoints implant revision for any reason and cup revision for aseptic reasons. An interaction term between underlying diagnosis and sex, and between underlying diagnosis and previous surgery, was incorporated in the model.

All competing risks analyses have been performed with the package mstate and cmprsk in the R software environment [15,16].

Violation of the proportional hazard assumptions were tested with Schoenfeld residuals with the function coxzph in the survival package. Sensitivity analysis was performed to study the effect of missing data on the outcome. All other statistical analyses were performed using SPSS 25 (IBM Corp, Armonk, NY, USA).

### Ethics, registration, data sharing, funding, use of AI, and disclosures

All data was pseudonymized before the LROI provided it. According to Dutch legislation this study is not subject to the Medical Research Involving Human Subjects Act (WMO), and we received a waiver from our local ethics review board (registration ID W.23.003, date March 13, 2023). Informed consent was not obtained. Data sharing is not possible. There was no specific source of funding for this study. All authors have no disclosures relevant to this study. Complete disclosure of interest forms according to ICMJE are available on the article page, doi: 10.2340/17453674.2024.41342

## Results

**Figure 1:**
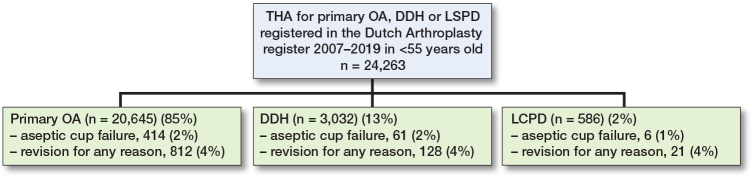
Flowchart showing the total number of THAs, the number per indication and the number of revisions for aseptic cup failure and for any reason.

We included 24,263 THAs (Figure 1). The registered indication for surgery was OA in 20,645 (85%), DDH in 3,032 (13%), and LCPD in 586 (2%) (Table 1). Mean age at primary arthroplasty was 49 years (SD 5.6) in the OA group, 44 (SD 8.9) in the DDH group, and 40 (SD 10.4) in the LCPD group.

**Table 1:** Baseline characteristics. Values are count (%)

Factor	OA	DDH	LCPD	Overall
Sex				
Male	9,288 (45)	761 (25)	372 (63)	10,421
Unknown	182 (0.9)	3 (0.1)	1 (0.2)	186
Left side	9,799 (47)	1,455 (48)	305 (52)	11,559
Age at surgery, years				
< 30	292 (1.4)	270 (8.9)	118 (20)	680
30–39	933 (4.5)	500 (16)	132 (23)	1,565
40–49	7,312 (35)	1,359 (45)	225 (38)	8,896
≥ 50	11,834 (57)	899 (30)	111 (19)	12,844
Unknown	274 (1.3)	4 (0.1)	0 (–)	278
Previous surgery	1,377 (6.7)	914 (30)	202 (34)	2,493
Unknown	844 (4.1)	105 (3.5)	15 (2.6)	964
ASA class				
1	9,281 (45)	1,635 (54)	330 (56)	11,246
2	9,645 (47)	1,214 (40)	230 (39)	11,089
3–4	1,249 (6.0)	160 (5.3)	20 (3.4)	1,429
Unknown	470 (2.3)	23 (0.8)	6 (1.0)	499
BMI				
< 18.5	85 (0.4)	30 (1.0)	6 (1.0)	121
18.5–25	3,911 (19)	796 (26)	140 (24)	4,847
25– 30	5,544 (27)	724 (24)	131 (22)	6,399
30–40	3,731 (18)	463 (15)	93 (16)	4,287
> 40	260 (1.3)	18 (0.6)	8 (1.4)	286
Unknown	7,114 (34)	1,001 (33)	208 (35)	8,323
Smoking, yes	2,661 (13)	327 (11)	91 (16)	3,079
Unknown	7,520 (36)	1,070 (35)	217 (37)	8,807
Fixation type				
Uncemented	17,414 (84)	2,108 (70)	432 (74)	19,954
Hybrid	1,353 (6.6)	387 (13)	54 (9.2)	1794
Reverse hybrid	375 (1.8)	78 (2.6)	10 (1.7)	463
Cemented	1,310 (6.3)	435 (14)	84 (14)	1,829
Unknown	193 (0.9)	24 (0.8)	6 (1.0)	223
Acetabular cup fixation type				
Uncemented	17,789 (86)	2,186 (72)	442 (75)	20,417
Unknown	593 (2.9)	24 (0.8)	6 (1.0)	623
Surgical approach				
Anterior	4,727 (23)	353 (12)	74 (13)	5,154
Anterolateral	1,096 (5.3)	104 (3.4)	29 (4.9)	1,229
Straight lateral	2,927 (14)	449 (15)	94 (16)	3,470
Posterolateral	11,537 (56)	2,089 (69)	385 (66)	14,011
Other/unknown	358 (1.7)	37 (1.2)	4 (0.7)	399
Articulation type **^a^**				
ZoP	1,698 (8.2)	158 (5.2)	36 (6.1)	1,892
MoP	3,549 (17)	655 (22)	98 (17)	4,302
CoP	11,469 (56)	1,694 (56)	344 (59)	13,507
CoC	2,573 (12)	293 (9.7)	72 (12)	2,938
Unknown	1,356 (6.6)	232 (7.7)	36 (6.1)	1,624
Acetabular cup size, mm				
< 44	47 (0.2)	37 (1.2)	0 (-)	84
44–48	2,213 (11)	734 (24)	98 (17)	3,045
49–54	11,615 (56)	1,657 (55)	312 (53)	13,584
55–60	5,590 (27)	430 (14)	148 (25)	6,168
> 60	457 (2.2)	37 (1.2)	7 (1.2)	501
Unknown	723 (3.5)	137 (4.5)	21 (3.6)	881
Femoral head size, mm				
22–28	4,287 (21)	1,038 (34)	155 (26)	5,480
32	10,423 (50)	1,429 (47)	278 (47)	12,130
36	4,952 (24)	442 (15)	126 (22)	5,520
≥	271 (1.3)	37 (1.2)	8 (1.4)	316
Unknown	712 (3.4)	86 (2.8)	19 (3.2)	817

OA: osteoarthritis; DDH: developmental dysplasia of the hip; LCPD: Legg–Calvé-Perthes disease; ASA: American Society of Anesthesiologists; BMI: body mass index.

a ZoP: zirconium-on-polyethylene; MoP: metal-on-polyethylene; CoP: ceramic-on-polyethylene; CoC: ceramic-on-ceramic.

Previous surgery on the affected hip was less common in the OA group (7%) than in the DDH (30%) or LCPD (35%) group (Table 1). There were more males in the LCPD (64%) than in the OA (45%) or DDH (25%) group. The posterolateral approach was most commonly used in all groups.

The cup was more often uncemented in the OA group (86%) than in the DDH (72%) or LCPD (75%) groups. The median acetabular cup size was 52 mm in all groups (ranging from 37–80 mm in the OA group, 35–68 mm in the DDH group, and 44–64 mm in the LCPD group), and cup size varied between groups (Table 1).

23,055 patients were alive with their primary implant in situ (95%), and 436 patients (2%) were dead. The remaining patients underwent 1 or more revision procedures during the study period.

**Figure 2:**
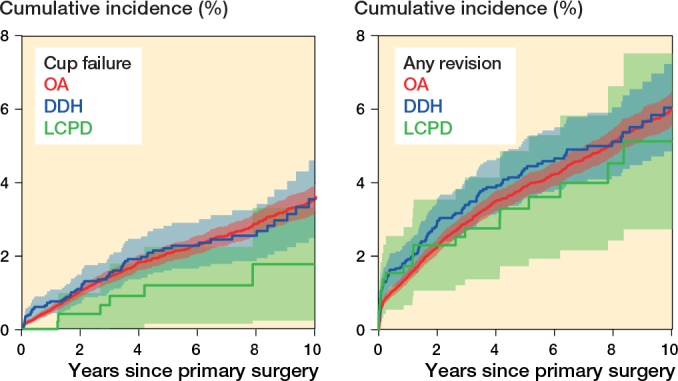
Cumulative incidence (%) of revision for aseptic cup failure (left panel) and of overall revision (right panel) according to underlying diagnosis. For Abbreviations, see Table 1.

The 10-year cumulative aseptic cup revision risk was 3.4% (CI 3.0–3.8) in the OA group, 3.4% (C, 2.4–3.4) in the DDH group, and 1.7% (CI 0.2-–3.1) in the LCPD group. The 10-year cumulative overall revision risks were 6.0% (CI 5.6-–6.5) in the OA group, 6.0% (CI 4.9–7.2) in the DDH group and 5.1% (CI 2.7–7.5) in the LCPD group, respectively (Table 2). The cumulative incidences for cup revision for aseptic reasons and for overall revision for each diagnosis are shown in Figure 2.

**Table 2:** Cumulative incidence (%) with 95% confidence interval (CI) of aseptic cup revision and overall revision risk, stratified according to underlying diagnosis

Factor Years since surgery	Cumulative incidence (%) (CI)		
	OA	DDH	LCPD
Aseptic cup revision			
2	1.0 (0.9–1.1)	1.2 (0.8–1.6)	0.4 (0.0–0.9)
5	2.0 (1.8–2.2)	2.1 (1.5–2.7)	1.1 (0.1–2.1)
10	3.4 (3.0–3.8)	3.4 (2.4–3.4)	1.7 (0.2–3.1)
Overall revision risk			
2	2.3 (2.1–2.5)	3.4 (2.7–4.1)	2.3 (1.1–3.5)
5	3.9 (3.6–4.2)	4.4 (3.6–5.2)	3.3 (1.7–4.9)
10	6.0 (5.6–6.5)	6.0 (4.9–7.2)	5.1 (2.7–7.5)

For Abbreviations, see Table 1.

In the multivariable Cox regression analysis for aseptic cup revision, the adjusted hazard ratio (HR) was 0.7 (CI 0.5–1.2) for DDH, and 0.8 (CI 0.3–2.1) for LCPD, respectively. For overall revision, the adjusted HR was 1.0 (CI 0.8–1.4) for DDH, and 1.2 (CI 0.7–2.1) for LCPD, respectively (Table 3). Interactions between underlying diagnosis and patient sex and underlying diagnosis and previous surgery were not significant and therefore were not incorporated in the final model. Sensitivity analysis provided the same conclusions concerning the HR for DDH and LCPD.

**Table 3:** Hazard ratios (HR) of Cox regression analyses with 10-year aseptic cup revision and overall revision for any reason as the endpoint for DDH and LCPD compared with the reference category primary OA

Analysis	Diagnosis	Aseptic cup revision HR (CI)	Revision for any reason HR (CI)
Univariate	OA	Ref.	Ref.
	DDH	1.0 (0.8–1.3)	1.1 (0.9–1.3)
	LCPD	0.5 (0.2–1.1)	0.9 (0.6–1.4)
Multivariate ^a^	OA	Ref.	Ref.
	DDH	0.7 (0.5–1.2)	1.0 (0.8–1.4)
	LCPD	0.8 (0.3–2.1)	1.2 (0.7–2.1)

For Abbreviations, see Table 1.

a In this model, age, patient sex, BMI, acetabular cup size, and previous surgery were used as covariates.

## Discussion

We aimed to investigate the 10-year cumulative aseptic cup revision and overall revision risk of THA for DDH and LCPD, and investigated whether these are increased compared with THAs for primary OA in patients below 55 years. The indication of a pediatric hip disorder was not associated with an increased risk of 10-year aseptic cup revision or overall revision risk, compared with THA performed for OA.

The risk of aseptic cup failure at 10 years in our study ranged from 1.7% to 3.4% for the 3 groups of underlying diagnoses. Few studies assessed the mid- to long-term aseptic cup failure risk in younger patients. Chougle et al. found a 9.4% risk of aseptic cup loosening in 292 cemented THAs for DDH [17]. Eskelinen et al. found > 90% survival rates for various uncemented cup designs in a study on 5,607 THAs performed for primary OA in patients aged younger than 55 [4]. A study from the Swedish Hip Arthroplasty Register on 8,043 THAs noted an increased risk of aseptic cup revision in patients under the age of 50 years [18]. Therefore, the aseptic cup revision risk we found seems to compare favorably with the literature.

The overall 10-year revision risk in our study ranged from 5.1% to 6.0%. Various studies found that younger patients have an increased revision risk after THA [1,19-22]. In the Finnish arthroplasty register, the 10-year survival rate in patients younger than 55 years was 72% for THA performed from 1980–1999 [23]. A systemic review found an estimated revision-free survival rate of 72–86% at 10 years for patients less than 60 years old [20]. A possible explanation for the favorable revision rates we found is that we included arthroplasties performed from 2007 onwards, while others included THAs performed earlier. The revision risk of THA has decreased over the last decades, presumably owing to improved implant designs and surgical technique [24,25].

There are a number of potential explanations for the fact that we found no statistically significant differences in revision risks between patients with primary OA and pediatric hip disorders. First, there is a risk of misclassification. On the one hand, the most severe DDH and LCPD cases are more likely to be classified in the correct group, and this may enhance the observed difference. On the other hand, less severe DDH and LCPD cases may be misclassified in the primary OA group, and these may cause an underestimation of the expected results. Although speculative, surgeons may have a higher threshold to revise more complex acetabular reconstructions in patients with DDH or LCPD if there is no strict indication for revision surgery, for example in cases of instability or progressive component loosening. Apart from that, patients with severe pediatric hip disorders and a long history of debilitating complaints are more likely to have low functional demands, which in turn may lead to a lower risk of mechanical failure, and these patients may be more hesitant to undergo revision surgery.

### Limitations

First, classification of the underlying hip disorder relied on the surgeon’s report, and could not be confirmed by radiographs or other means. Second, we were not aware of any underlying neurological disorders, such as cerebral palsy or meningomyelocele, while these conditions are associated with hip dysplasia and may influence the outcome and revision risk of THA [26,27]. However, these underlying causes are rare, therefore they are not likely to substantially influence the outcomes of our study [28]. Third, missing data is inherent to the use of register data. Although the reporting completeness of our national register has always been high and has further improved over recent years, it is likely that we have missed a number of patients, and not all data was complete for all patients. However, it is unlikely this has influenced our results and conclusions, as the numbers of missing data are limited and the data is most likely missing at random.

### Conclusions

We found minor increased overall risk of revisions at 2 years but not of aseptic cup revision or overall revision at 5 and 10 years, for THA performed under the age of 55 years for a pediatric hip disorder, compared with primary OA.

In perspective, however, pediatric hip disorders may result in specific surgical challenges when performing THA and we are of the opinion that surgeons should be hesitant to perform THA in young patients, given the increased lifetime revision risk.

## Notes

MB and PBdW wrote the original draft. Conceptualization was performed by PBdW. Formal analyses were performed by MB and MF. Methodology was done by MB, MG, MF, and PBdW. Supervision was done by PBdW, MG, and RN. All authors critically reviewed the final manuscript.

Handling co-editors: Keijo Mäkelä and Philippe Wagner

*Acta* thanks Antti Eskelinen and David F Hamilton for help with peer review of this manuscript.
